# Natural History, Phenotypic Spectrum, and Discriminative Features of Multisystemic RFC1 Disease

**DOI:** 10.1212/WNL.0000000000011528

**Published:** 2021-03-02

**Authors:** Andreas Traschütz, Andrea Cortese, Selina Reich, Natalia Dominik, Jennifer Faber, Heike Jacobi, Annette M. Hartmann, Dan Rujescu, Solveig Montaut, Andoni Echaniz-Laguna, Sevda Erer, Valerie Cornelia Schütz, Alexander A. Tarnutzer, Marc Sturm, Tobias B. Haack, Nadège Vaucamps-Diedhiou, Helene Puccio, Ludger Schöls, Thomas Klockgether, Bart P. van de Warrenburg, Martin Paucar, Dagmar Timmann, Ralf-Dieter Hilgers, Jose Gazulla, Michael Strupp, German Moris, Alessandro Filla, Henry Houlden, Mathieu Anheim, Jon Infante, A. Nazli Basak, Matthis Synofzik, on behalf of the RFC1 Study Group

**Affiliations:** From the Department of Neurodegenerative Diseases (A.T., S.R., L.S., M. Synofzik), Hertie-Institute for Clinical Brain Research and Center of Neurology, and German Center for Neurodegenerative Diseases (DZNE) (A.T., S.R., L.S., M. Synofzik), University of Tübingen, Germany; MRC Centre for Neuromuscular Diseases (A.C., N.D., H.H.), Department of Neuromuscular Diseases, National Hospital for Neurology and Neurosurgery, UCL Queen Square Institute of Neurology, London, UK; Department of Brain and Behaviour Sciences (A.C.), University Pavia, Italy; Department of Neurology (J.F., T.K.), University Hospital Bonn; German Center for Neurodegenerative Diseases (DZNE) (J.F., H.J., T.K.), Bonn; Department of Neurology (H.J.), University Hospital of Heidelberg; Department of Psychiatry, Psychotherapy and Psychosomatics (A.M.H., D.R.), University of Halle, Germany; Département de Neurologie (S.M., M.A.), Hôpital de Hautepierre, Hôpitaux Universitaires de Strasbourg; Department of Neurology (A.E.-L.), APHP, CHU de Bicêtre; French National Reference Center for Rare Neuropathies (NNERF) (A.E.-L.); Inserm U1195 and Paris-Sud University (A.E.-L.), Le Kremlin Bicêtre, France; Medical Faculty (S.E.), Department of Neurology, Uludag University, Bursa, Turkey; University of Zurich (V.C.S., A.A.T.); Department of Neurology (V.C.S., A.A.T.), University Hospital Zurich, Switzerland; Institute of Medical Genetics and Applied Genomics (M. Sturm, T.B.H.) and Center for Rare Diseases (T.B.H.), University of Tübingen, Germany; Institut de Génétique et de Biologie Moléculaire et Cellulaire (IGBMC) (N.V.-D., H.P.); INSERM (N.V.-D., H.P.), U1258; CNRS (N.V.-D., H.P.), UMR7104, Illkirch; Université de Strasbourg (H.P.), France; Department of Neurology (B.P.v.d.W.), Donders Institute for Brain, Cognition and Behaviour, Radboud University Medical Centre, Nijmegen, the Netherlands; Department of Neurology (M.P.), Karolinska University Hospital; Department of Clinical Neuroscience (M.P.), Karolinska Institute, Stockholm, Sweden; Department of Neurology (D.T.), Essen University Hospital, University of Duisburg-Essen, Essen; Department of Medical Statistics (R.-D.H.), RWTH Aachen University, Germany; Department of Neurology (J.G.), Hospital Universitario Miguel Servet. Zaragoza, Spain; Department of Neurology (M. Strupp), University Hospital, and German Center for Vertigo and Balance Disorders (M.Strupp), Ludwig Maximilians University, Munich, Germany; Neurology Service (G.M.), Hospital Unversitario Central de Asturias (HUCA), SESPA, Oviedo, Spain; Department of Neurosciences and Reproductive and Odontostomatological Sciences (A.F.), Federico II University Naples, Italy; Institute of Genetics and Molecular and Cellular Biology (M.A.), INSERM-U964/CNRS-UMR7104, University of Strasbourg, Illkirch; Strasbourg Federation of Translational Medicine (M.A.), University of Strasbourg, Strasbourg, France; Service of Neurology (J.I.), University Hospital “Marqués de Valdecilla (IDIVAL),” University of Cantabria, “Centro de Investigación Biomédica en Red de Enfermedades Neurodegenerativas (CIBERNED),” Santander, Spain; and Suna and Inan Kıraç Foundation (A.N.B.), Neurodegeneration Research Laboratory, KUTTAM, Koç University School of Medicine, Istanbul, Turkey.

## Abstract

**Objective:**

To delineate the full phenotypic spectrum, discriminative features, piloting longitudinal progression data, and sample size calculations of replication factor complex subunit 1 (RFC1) repeat expansions, recently identified as causing cerebellar ataxia, neuropathy, vestibular areflexia syndrome (CANVAS).

**Methods:**

Multimodal *RFC1* repeat screening (PCR, Southern blot, whole-exome/genome sequencing–based approaches) combined with cross-sectional and longitudinal deep phenotyping in (1) cross-European cohort A (70 families) with ≥2 features of CANVAS or ataxia with chronic cough (ACC) and (2) Turkish cohort B (105 families) with unselected late-onset ataxia.

**Results:**

Prevalence of RFC1 disease was 67% in cohort A, 14% in unselected cohort B, 68% in clinical CANVAS, and 100% in ACC. RFC1 disease was also identified in Western and Eastern Asian individuals and even by whole-exome sequencing. Visual compensation, sensory symptoms, and cough were strong positive discriminative predictors (>90%) against RFC1-negative patients. The phenotype across 70 RFC1-positive patients was mostly multisystemic (69%), including dysautonomia (62%) and bradykinesia (28%) (overlap with cerebellar-type multiple system atrophy [MSA-C]), postural instability (49%), slow vertical saccades (17%), and chorea or dystonia (11%). Ataxia progression was ≈1.3 Scale for the Assessment and Rating of Ataxia points per year (32 cross-sectional, 17 longitudinal assessments, follow-up ≤9 years [mean 3.1 years]) but also included early falls, variable nonlinear phases of MSA-C–like progression (SARA points 2.5–5.5 per year), and premature death. Treatment trials require 330 (1-year trial) and 132 (2-year trial) patients in total to detect 50% reduced progression.

**Conclusions:**

RFC1 disease is frequent and occurs across continents, with CANVAS and ACC as highly diagnostic phenotypes yet as variable, overlapping clusters along a continuous multisystemic disease spectrum, including MSA-C-overlap. Our natural history data help to inform future RFC1 treatment trials.

**Classification of Evidence:**

This study provides Class II evidence that RFC1 repeat expansions are associated with CANVAS and ACC.

Biallelic intronic AAGGG repeat expansions in replication factor complex subunit 1 (*RFC1)* have recently been described as a frequent cause of late-onset ataxia, especially in cerebellar ataxia, sensory neuropathy, and vestibular areflexia syndrome (CANVAS).^1–4^ An estimated allele frequency of 0.7% to 4%,^1,2^ as in the range of Friedreich ataxia, suggests that pathogenic *RFC1* repeat expansions cause unrecognized phenotypes of a more common disease,^5^ indicates a significant number of as-yet unidentified RFC1 patients, and highlights the need to prepare first translational steps toward trial readiness for this novel disease.

While a recent study has provided first insights into the phenotype and evolution of the disease by cross-sectional data,^5^ confirmation from an independent large-scale cohort but, in particular, in-depth longitudinal phenotyping and quantitative natural history data on RFC1 disease are warranted. To prepare future treatment trials in RFC1 disease, we here leveraged a large cross-European multicenter ataxia cohort to (1) map its full phenotypic spectrum and evolution beyond CANVAS (using both a cohort expected to be enriched for RFC1 disease and an independent cohort of unselected patients with late-onset ataxia); (2) single out discriminative features of patients with RFC1-positive and RFC1-negative ataxia; and (3) map the natural disease history, including first piloting quantitative longitudinal disease progression data and preliminary sample size calculations.

## Methods

### Primary Research Question

The primary aims of our study were to screen for *RFC1* repeat expansions in patients with features of CANVAS or ACC and to delineate the full phenotypic spectrum, discriminative features, and progression of RFC1 disease. Given its double cohort design, this study provides Class II evidence that *RFC1* repeat expansions are associated with CANVAS and ACC.

### Patient Cohorts and Recruitment

Patients were recruited from 2 independent screening cohorts. Cohort A was designed to be likely enriched for RFC1-positive patients according to the phenotypic selection criteria, namely unsolved degenerative ataxia and at least 2 phenotypic features of CANVAS or ataxia with chronic cough^6^ (ACC), that is, cerebellar ataxia, sensory neuropathy, vestibulopathy, or chronic cough (defined as an otherwise unexplained cough persisting >8 weeks).^7^ Here, we aggregated 76 deep-phenotyped patients from 70 families from 14 different sites in Europe (France 13, Germany 45, Italy 2, the Netherlands 2, Sweden 1, Spain 9, Switzerland 4). This cohort had undergone exclusion of SCA1/2/3 in 49%, Friedreich ataxia in 47%, and negative whole-exome sequencing (WES) or targeted sequencing panel in 34% of patients. Fifty-nine patients of this cohort A were classified as having CANVAS, regardless of the additional presence of cough (in a subset of 19 of 59 patients), and 29 patients were classified as having ACC, regardless of additional vestibulopathy (in a subset of 19 of 29 patients). Cohort B served as an independent test cohort of consecutive unselected patients with late-onset ataxia from a major diagnostic referral center in Turkey (Bosporus University/Koç University), representing the diagnostic yield of RFC1-positive patients under conditions of unbiased daily “as-comes-in” diagnostic routine. Here, we screened 105 index patients with late-onset recessive or sporadic cerebellar ataxia (age range 38–71 years, 50% consanguinity, exclusion of SCA1/2/3 in 44% and Friedreich ataxia in 33%, negative WES in 10% of patients), without any further phenotypic selection criteria. At the screening stage, phenotypic information in cohort B was limited to the mere information provided on the genetic test request forms (cerebellar ataxia only 75, cerebellar ataxia and neuropathy 19, cerebellar ataxia and vestibulopathy 8, CANVAS 3).

### Deep Phenotyping

For all patients in cohort A (including 6 previously published^6,8^) and all RFC1-positive patients in cohort B, patients or records were systematically reassessed by the local physician according to a common comprehensive standardized data sheet developed by our RFC1 study group (supplement 1, doi.org/10.5061/dryad.1vhhmgqrd). All patients had at least 1 neurologic examination; longitudinal data with ≥2 prospective examinations were available from 31 patients. Classification as CANVAS required clinical evidence of cerebellar, neuropathic (abnormal vibration sense with or without abnormal ankle reflex^8^), and bilateral vestibular (vestibulo-ocular reflex by head impulse test or video-oculography) damage. In contrast to the formal diagnostic criteria of CANVAS,^3^ clinical rather than electrophysiologic criteria were taken, for example, for sensory neuropathy, thus allowing us to increase the sensitivity of our screening and capture patients from the various centers across Europe. The phenotype was classified as multisystemic if any additional feature was present in addition to the features of the CANVAS systems, for example, hypokinetic or hyperkinetic movement disorders, pyramidal signs, slow saccades, cognitive impairment, or signs of autonomic dysfunction. Ataxia severity was assessed by the Scale for the Assessment and Rating of Ataxia (SARA).^9^ Functional impairment was rated with the Spinocerebellar Degeneration Functional Score (SDFS) on the basis of patient history and clinical examinations.^10^

### Standard Protocol Approvals, Registrations, and Patient Consents

This study has been approved by the Institutional Review Board of the University of Tübingen (Az. 598/2011BO1), and all patients or legal representatives provided informed consent according to local regulations.

### Genetic Screening for *RFC1* Repeat Expansions

**Figure 1:**
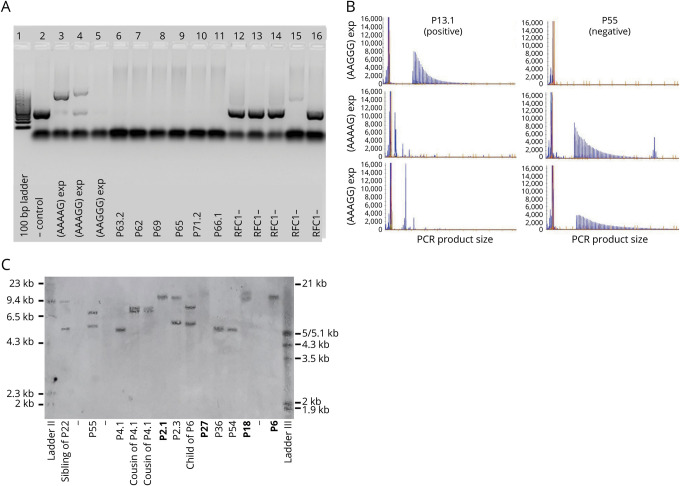
Identification of RFC1 Repeat Expansions (A) Flanking PCR, products run on a 1% agarose gel. Lanes 2 to 5: controls, including reference sequence (AAAAG)_11_ in lane 2. Lanes 6 to 11: positive samples with absent product amplification indicating biallelic pathogenic AAGGG expansions. Lanes 12 to 16: negative samples with product amplification consistent with reference sequence or a nonpathogenic expansion. (B) Repeat-primed PCR targeting the pathogenic (AAGGG)_exp_, nonpathogenic (AAAAG)_exp_, and (AAAGG)_exp_ expansions; visualization of separated fluorescein amidite–labeled PCR products. Ladder markers are 35, 50, 75, 100, 139, 150, 160, 200, 250, 300, 340, 350, 400, 450, 490, and 500 nucleotides. Representative plots from a P13.1 carrying the biallelic AAGGG repeat expansion and P55 carrying nonpathogenic (AAAGG)exp/(AAAAG)exp expansions in compound heterozygous state. (C) Southern blotting of genomic DNA from 13 patients and unaffected relatives. Patients carrying biallelic pathogenic expansions in *RFC1* (bold) show 2 discrete or 1 overlapping band. In RFC1-negative cases, either one 5-kb band corresponding to the expected size for reference allele (AAAAG)_11_ or bands of increased size can be observed.

**Figure 2:**
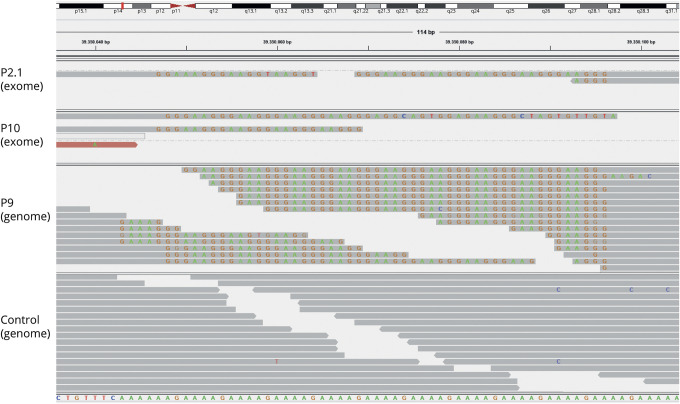
RFC1 Screening by Next-Generation Sequencing Integrative genomics viewer visualization of exome (P2.1, P10) and genome (P9) sequencing reads aligned to the repeat locus and flanking regions showing the presence of a mutated AAGGG repeat motif. Sequence reads from both directions are interrupted and do not span the entire length of the repeat expansion. The repeated AAGGG motif does not map to the (AAAAG)_11_ reference sequence, and reads showing the sequence alteration are soft-clipped. The lower panel shows sequence reads from a control genome dataset. RFC1 = replication factor complex subunit 1.

Genetic screening for *RFC1* repeat expansions was performed in all index patients and in all affected and unaffected family members for whom DNA was available with the use of an established stepwise *RFC1* repeat sequencing and confirmation procedure as previously described (figure 1, A–C).^1^ In a small independent exploratory analysis, we aimed to investigate whether the intronic AAGGG repeat motif can also be detected in whole-genome sequencing (WGS) and even WES datasets. We developed a data screening algorithm that first searched for sequence reads mapping within the chromosomal position of the *RFC1* repeat expansion and then displayed the AAGGG motif in soft-clipped reads, in WES leveraging unintended off-target reads incidentally overlapping the repeat locus (figure 2; for details, see supplement 2, doi.org/10.5061/dryad.1vhhmgqrd; manuscript in preparation). This approach was paradigmatically applied to 1 WGS (P9) and 2 WES (P2.1, P10) datasets for which research consent was available.

### MRI Imaging

Findings from routine brain MRI were systematically aggregated from all patients in whom such MRI results were available. In addition, digital routine brain MRIs including T1-, T2-, and diffusion-weighted images and fluid-attenuated inversion recovery T2 images were systematically assessed by 2 central independent raters (M.S., A.T.) when such images were available and digitally transferable for centralized review.

### Statistical Analysis

All statistics were calculated with GraphPad Prism 8 (GraphPad Software, La Jolla, CA), SPSS 25 (IBM Corp, Armonk, NY), and SAS version 9.4 (SAS Institute Inc, Cary, NC), using 2-sided tests with significance set at *p* < 0.05. Cross-sectional annual disease progression was estimated by the ratio of each participant’s ataxia severity (last SARA score) and ataxia duration.^11,12^ Longitudinal annualized disease progression was estimated with the linear mixed-effect modeling restricted-maximum-likelihood method with random effects on intercept and slope (proc MIXED in SAS).^13^ From this linear mixed-effect modeling estimate, we calculated sample sizes that would enable the detection of variable reductions in SARA score progression in parallel-group interventional trials with 3 visits in observation periods of either 1-year (0, 6, and 12 months) or 2-year (0, 12, and 24 months) duration. Phenotypic features characterizing ataxia and neuropathy and multisystemic features observed with high prevalence (>10%) in the RFC1-positive cohort were compared between RFC1-positive and RFC1-negative patients with the use of parametric *t* tests, nonparametric Mann-Whitney *U* test, and Fisher exact test for proportions as required by the data. We calculated 95% confidence intervals (CIs) of frequency estimates using the adjusted Wald method to account for small groups.

### Data Availability

Deidentified data supporting the findings of this study (including single-participant data) are provided in supplement 1 (doi.org/10.5061/dryad.1vhhmgqrd). No consent has been obtained for open sharing of raw genetic or MRI data.

## Results

### Genetic Screening for *RFC1* Repeat Expansions

Biallelic AAGGG *RFC1* repeat expansions were identified in 52 patients from 47 of 70 families (67%, 95% CI 56%–77%) of cohort A with ≥2 clinical features of CANVAS or ACC and in 18 patients from 15 of 105 families (14%, 9%–22%) of the consecutive cohort B with unselected late-onset ataxia (for exemplary illustrations of PCR results, see figure 1, A and B). A screen for conventional (i.e., nonrepeat) *RFC1* mutations did not reveal any biallelic loss-of-function variants in *RFC1* in the WES/WGS datasets available (n = 10 datasets).

Overall, the 70 RFC1-positive patients originated from at least 12 different countries (see supplement 1 for patient details, along with *RFC1* repeat conformations and available sizes, doi.org/10.5061/dryad.1vhhmgqrd), including Eastern (Indonesia) and Western Asian (Asia Minor, Anatolia) origins. Twenty patients from 15 RFC1-positive families (27%) had probable consanguineous parents, and 18 families (32%) had a multiplex family history. Segregation analysis, performed in a total of 38 first-degree relatives (3 parents, 3 children, 32 siblings) for whom DNA was available, demonstrated perfect segregation of biallelic variants for an autosomal-recessive inheritance, with 2 pathogenic repeat expansions in 9 of 10 affected relatives, and no or only 1 pathogenic repeat expansion in all 28 healthy relatives. The only exception was patient P2.3 and sister of twin P2.1/2.2, who showed a clinical syndrome of CANVAS but carried only 1 heterozygous *RFC1* expansion. Clinical reassessment indicated that neuropathy, vestibulopathy, and cerebellar ataxia (which was only very mild) in this patient was secondary to prior chemotherapies with epirubicin, docetaxel, and cyclophosphamide. Southern blot, performed for a subset of participants for whom sufficient DNA amounts were available, confirmed the presence of the biallelic pathogenic repeat expansion in all 18 of 18 investigated patients with a positive PCR and the absence of the biallelic repeat expansion in all 10 of 10 investigated patients and 6 of 6 relatives with a negative PCR or a carrier status (for exemplary illustrations of Southern blot results, see figure 1C).

In a parallel feasibility approach, we exemplarily explored whether a next-generation sequencing–based approach also might allow detecting AAGGG repeat motifs, both in WGS (1 patient, P9) and—despite the intronic location of the *RFC1* repeat—in WES (2 patients, P2.1 and P10) (figure 2). In the WGS dataset (P9), multiple aberrant sequence reads were identified. In line with a homozygous state, visualization demonstrated that all sequencing reads carried the soft-clipped AAGGG motif but no wild-type sequences. In the WES datasets, 3 reads (P2.1) and 2 reads (P10) indicated mutated sequences. The presence of homozygous single nucleotide polymorphisms in adjacent coding regions helped to further indicate and support the presence of homozygous repeat expansions in these WES datasets. The *RFC1* changes were confirmed in all 3 participants by the independent, conventional PCR-based approach.

### CANVAS and ACC in RFC1 Disease

**Figure 3:**
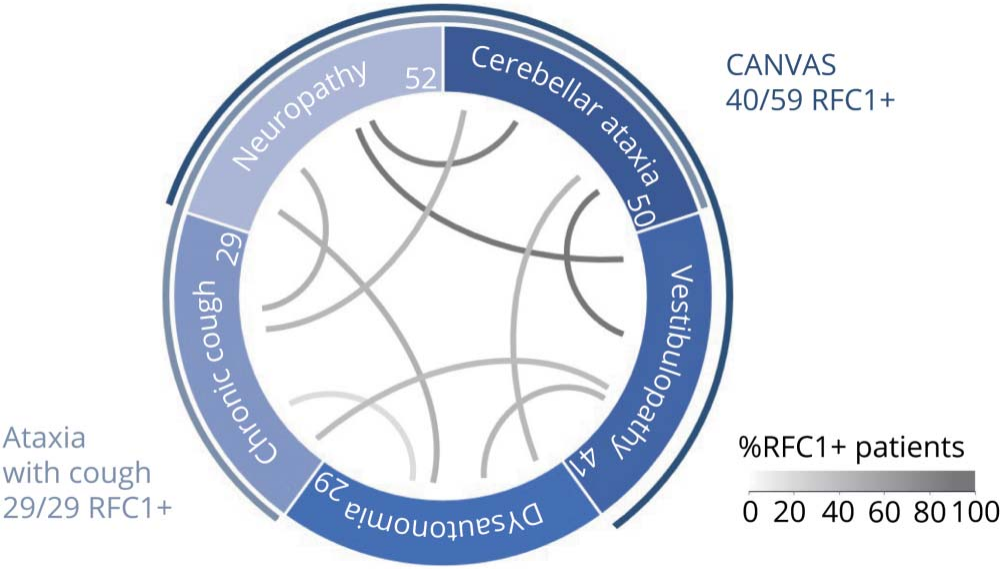
Continuous Spectrum of Variable Feature Combinations in RFC1 Disease Prevalence of key features of replication factor complex subunit 1 (RFC1) disease and their within-participant combinations in 52 RFC1-positive patients (all from cohort A). Absolute number of patients with individual feature shown in circle sections. Lines represent relative co-occurrence of 2 features among RFC1-positive patients with reported presence or absence of both features. Within this combinatorial spectrum, the combination of ataxia with chronic cough and the triad of cerebellar ataxia, neuropathy, and vestibulopathy areflexia syndrome (CANVAS) represent just 2 instances of variable clusters along a continuous overlapping spectrum of RFC1 disease yet with a relatively increased associative strength.

Biallelic pathogenic *RFC1* repeat expansions were found in all 29 patients with ACC in cohort A (100%, 95% CI 90%–100%), demonstrating that *RFC1* is the major gene underlying this syndromic cluster. Biallelic pathogenic *RFC1* expansions were also found in 40 of 59 patients (68%, 95% CI 55%–78%) with clinically defined CANVAS and in 26 of 29 patients with CANVAS (90%, 95% CI 73%–98%) with additional electrophysiologic evidence,^3^ indicating that, while RFC1 is the major cause of CANVAS, other causes for CANVAS remain to be identified. When ACC and CANVAS are deconstructed into their constituent and frequently associated features (cerebellar ataxia, neuropathy, vestibulopathy, cough, and autonomic dysfunction^14^), analysis of the combinations of these single features in the 52 RFC1-positive patients from the deep-phenotyping cohort A shows that ACC and CANVAS do not occur as strictly delineated syndromic entities but rather as phenotypic clusters along a continuum of variable phenotypic combinations (figure 3).

### Multisystemic Spectrum of RFC1 Disease

**Figure 4:**
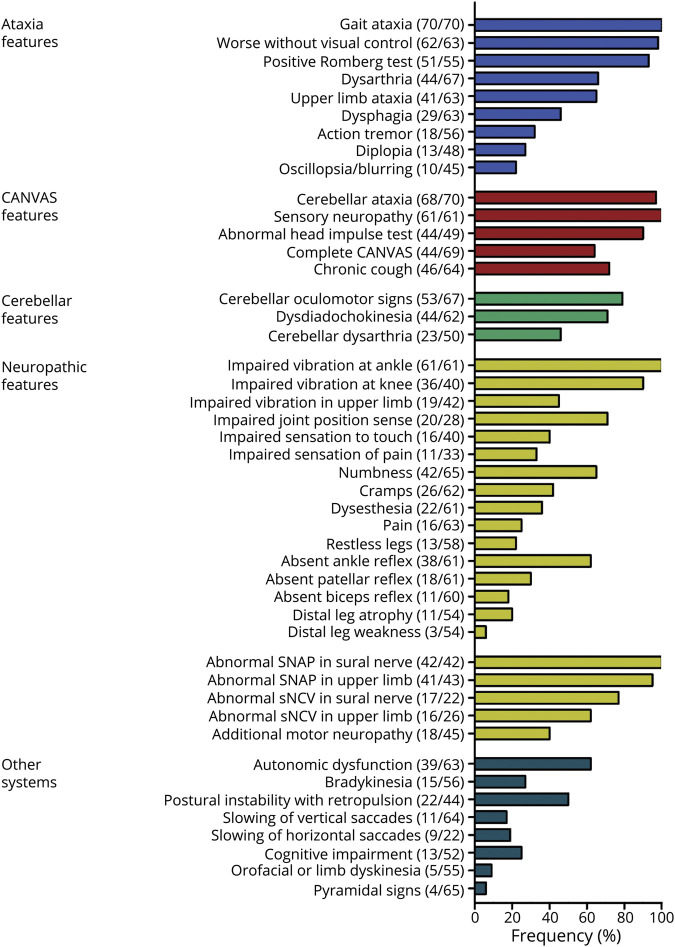
Phenotypic Spectrum of RFC1 Disease Prevalence of signs, symptoms, and electrodiagnostic findings of 70 patients with biallelic *RFC1* repeat expansions. Numerator and denominator in brackets indicate the number of affected patients and the number of patients assessed for this feature, respectively. CANVAS = cerebellar ataxia, neuropathy, and vestibular areflexia syndrome; RFC1 = replication factor complex subunit 1.

Given this heterogeneous multisystemic phenotype of RFC1, we analyzed individual phenotypic features across all 70 RFC1-positive patients from cohorts A and B (last examination a median of 11 years after disease onset, interquartile range [IQR] 8–17, range 0–26) in detail. Gait ataxia with clinical evidence of sensory ataxia, that is, marked worsening without visual control (98%) or a positive Romberg test (93%), was the main feature of RFC1 disease. Characteristics of cerebellar features, neuropathy, vestibulopathy, and chronic cough were consistent with those of a previous cohort^5^ (see figure 4 for details).

In 48 patients (69%), however, this mixed sensory-cerebellar ataxia was part of a broader multisystemic phenotype, including features overlapping with other neurodegenerative diseases (figure 4). Autonomic dysfunction (62%) and bradykinesia (28%, including 1 patient [P52] with levodopa response) were prevalent features overlapping with cerebellar-type multiple system atrophy (MSA-C), co-occurring in 13 patients (19%). Autonomic dysfunction included erectile dysfunction (44% of men), urinary urge or retention (39% of all patients), postural hypotension (23%), chronic constipation (24%), and even fecal incontinence (11%). Gastroesophageal reflux disease was present in 19 patients (31%) and was not significantly more prevalent in patients with cough than in those without cough (40% vs 19%, Fisher exact test, *p* = 0.211). REM sleep behavior disorder was reported in 3 patients (6%), and sleep apnea was reported by 7 (14%), again indicating overlap with MSA-C. Postural instability with retropulsion (49%) and slowing of vertical saccades (17%) co-occurred together with bradykinesia in 6 patients (9%), suggesting overlap also with progressive supranuclear palsy (PSP). Horizontal saccades were slow in 19% of patients. Hyperkinetic movement disorders (see video 1, links.lww.com/WNL/B309 and video 2, links.lww.com/WNL/B310)—namely orofacial dyskinesia (5%), orofacial dystonia (5%), or limb chorea (2%)—indicated some overlap with Huntington disease, with any of these 3 features occurring in 6 of 57 patients (11%). Mild cognitive impairment with mental slowing was reported in 25% of patients, whose age and disease duration were not different from those of patients without cognitive impairment (66.5 ± 9.6 vs 65.5 ± 9.5 and 11.8 ± 4.6 vs 12.4 ± 6.8, respectively, *t* test, both *p* > 0.726), indicating that it is not just an age-related or late-stage disease feature. While brisk reflexes were observed in 14%, prominent pyramidal tract involvement (spasticity or extensor plantar response), a common feature in the >60 other autosomal-recessive ataxias,^15^ was observed in only 4 patients (6%).

10.1212/011528_Video_1 Video 1 Movement disorders of P1.Download Supplementary Video 1 via http://dx.doi.org/10.1212/011528_Video_1

10.1212/011528_Video_2 Video 2 Movement disorders of P11-2.Download Supplementary Video 2 via http://dx.doi.org/10.1212/011528_Video_2

### Discriminative Features of RFC1-Positive Patients

Of all features, chronic cough, sensory ataxia with use of visual control, sensory neuropathy symptoms, and axonal sensory neuropathy yield the highest predictive values to discriminate RFC1-positive (n = 70) from RFC1-negative (n = 24) patients (table).

**Table:**
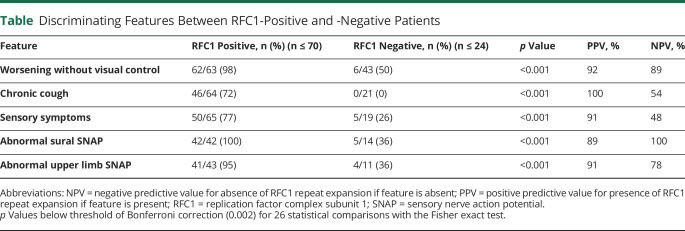
Discriminating Features Between RFC1-Positive and -Negative Patients

### Magnetic Resonance Imaging

**Figure 5:**
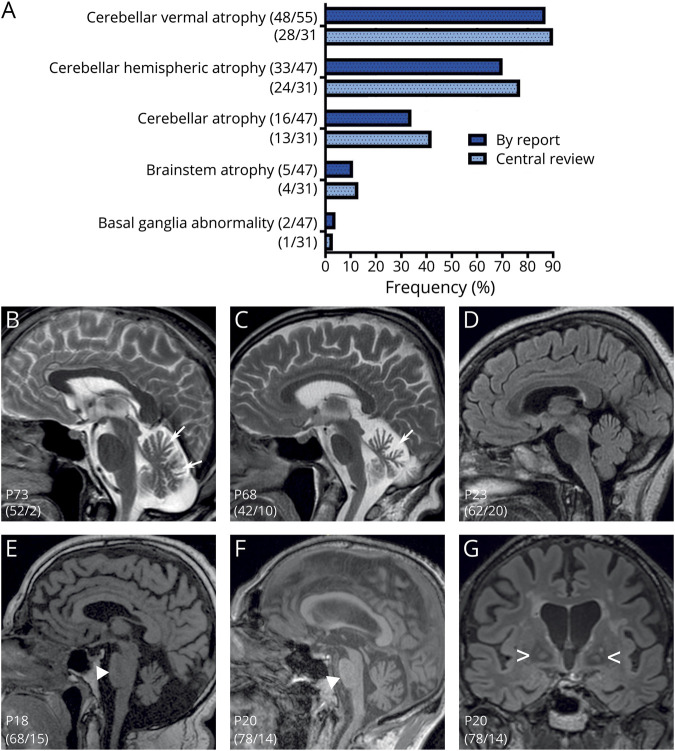
MRI Features of RFC1 Disease (A) MRI findings by report and by centralized review of digital images by 2 independent raters. Numerator and denominator in brackets indicate the number of patients with a feature and the number of patients assessed for this feature, respectively. (B–D) Mild to moderate cerebellar atrophy of the vermis with variable extent and temporal evolution, for example, marked cerebellar atrophy at 42 years of age in P68 vs absence of atrophy in P23 at 62 years of age after 20 years of ataxia. (E and F) Representative images of mild pontine atrophy after >14 years’ disease duration. (G) Patient with pallidal T2 signal abnormalities. Numerator and denominator in brackets on images indicate age and ataxia duration at MRI. RFC1 = replication factor complex subunit 1.

Analysis of brain MRI was based on reported findings for 56 RFC1-positive patients, complemented by a centralized rereview of digital images by 2 independent raters in 31 patients (median disease duration at MRI 10 years, range 0–23 years) (figure 5A). Cerebellar atrophy was the most prevalent finding but was not universal, affecting the cerebellar vermis more than the cerebellar hemispheres (87% and 70%, respectively, by MRI reports). While it was mild to moderate in most patients (89%) (figure 5B), patient P68 showed severe cerebellar atrophy already at 42 years of age after 10 years’ disease duration (figure 5C), and patient P23 exhibited no cerebellar atrophy at 62 years of age after 20 years' disease duration (figure 5D), thus highlighting the variable extent and temporal evolution of cerebellar atrophy in RFC1 disease. Disease duration did not differ between patients with and those without cerebellar atrophy (9.9 ± 4.7 vs 13.3 ± 11.6, *t* test, *p* = 0.658).

Cerebral atrophy (predominantly parietal) was reported in 37% of patients and detected in 42% of reviewed MRIs. Age at MRI (65.0 ± 9.8 vs 58.3 ± 6.8 years, *t* test, *p* = 0.031), but not disease duration at MRI (11.0 ± 5.7 vs 9.7 ± 5.4 years, *p* = 0.511), was higher in patients with cerebral atrophy, indicating that this MRI feature either might reflect accelerated age-related effects in RFC1 disease or is related to aging per se. In contrast, mild brainstem atrophy (pons, mesencephalon), which was detected in 13% of reviewed MRIs (figure 5, E and F), was associated with disease duration (17.0 ± 4.1 vs 9.2 ± 4.9 years, *p* = 0.006) but only borderline with age (68.8 ± 7.8 vs 60.0 ± 8.4 years, *p* = 0.058), suggesting a primarily direct disease-related feature. Clinically, brainstem atrophy was associated with increased frequency of dysphagia (100% vs 43% in patients without brainstem atrophy, Fisher exact test, *p* = 0.022), urinary urge or retention (80% vs 25%, *p* = 0.028), slowing of vertical saccades (60% vs 12%, *p* = 0.029) and horizontal saccades (60% vs 14%, *p* = 0.042), and increase of saccadic latency (80% vs 7%, *p* = 0.001). Signal abnormalities of basal ganglia were reported in 2 cases, including 1 case with a central review of MRI showing pallidal T2-signal elevation (figure 5G).

### Feature Evolution and Disease Progression

**Figure 6:**
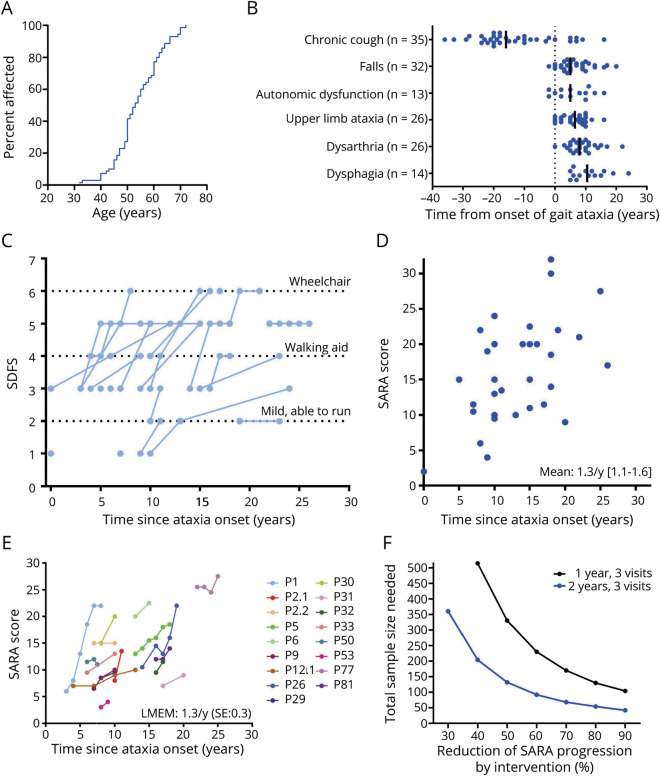
Feature Evolution and Disease Progression (A) Onset of ataxia in replication factor complex subunit 1 disease is relatively late (compared to other recessive ataxias),^26^ with 50% of patients affected by 54 years of age. (B) Temporal evolution of ataxia and nonataxia features relative to onset of gait ataxia (dotted line). (C) Cross-sectional and longitudinal functional disease progression as indicated by the Spinocerebellar Degeneration Functional Score (SDFS; n = 33). (D) Cross-sectional progression of ataxia indicated by the individual Scale for the Assessment and Rating of Ataxia (SARA) score at the last assessment relative to ataxia duration (n = 32). (E) Prospective longitudinal progression of ataxia (n = 17). Comparable ataxia severities (e.g., P32 vs P50, or P9 vs P31) and phases of accelerated progression (e.g., P1, P26) occur after interindividually variable durations of ataxia. (F) Sample size estimations for the detection of reduced SARA score progression in parallel-group (1:1) interventional trials with 3 visits in observation periods of either 1-year (0, 6, and 12 months) or 2-year (0, 12, and 24 months) duration. LMEM = linear mixed effect model; SE = standard error.

Onset of gait ataxia in patients with *RFC1* repeat expansions occurred at a median age of 53 years (range 32–72 years, IQR 49–60 years) (figure 6A) and was earlier in patients with chronic cough (n = 46, age 50.3 ± 7.3 years) than without chronic cough (n = 19, age 61.7 ± 7.4 years, *t* test, *p* < 0.001). Onset of chronic cough was at a median age of 35 years (n = 35; range 16–69 years, IQR 30–42 years) and preceded gait ataxia in 28 of 35 patients (80%) by up to 36 years (median −16 years) (figure 6B). Autonomic dysfunction (5 years, IQR 1–11 years), upper limb ataxia (6.5 years, IQR 2–10 years), dysarthria (8 years, IQR 6–11 years), and dysphagia (10 years, IQR 6–15 years) all occurred within ≤10 years after onset of gait ataxia, demonstrating that these autonomic, cerebellar, and brainstem dysfunctions are relatively early features of RFC1 disease. Similarly, first falls also already occurred after a median of 5 years (IQR 3–10 years) and were reported already as early as 2 years before ataxia onset (figure 6B). Walking aids were first required after a median of 10 years (n = 22, IQR 6–12 years).

A more specific analysis of this using cross-sectional and longitudinal SDFS scores (available for 33 patients) indicated dependence on walking aids (SDFS score 4–5) in 38% (3 of 8) within 5 years, 53% (9 of 17) within 10 years, and 68% (13 of 19) beyond 15 years of ataxia duration, suggesting a substantial interindividual variability in disease progression (figure 6C). Wheelchair dependence (SDFS score 6) occurred predominantly after >15 years of ataxia (4 of 19 patients). Premature death related to RFC1 disease (i.e., severe dysphagia with cachexia, cough, and immobility/bedridden; no evidence of any other secondary cause of death) occurred in patient P11.2 at 63 years of age after 22 years of disease and in P61 at 65 years of age already after 13 years of disease.

Cross-sectional progression of ataxia, based on SARA scores available for 32 patients, showed a moderate mean progression rate of 1.3 SARA points per year of ataxia (95% CI 1.1–1.6) (figure 6D). This cross-sectional estimate was corroborated by longitudinal data (available for 17 patients from 6 sites, 35 follow-up visits up to 9 years [median 3.1 years]), demonstrating a progression rate of 1.3 SARA points per year (standard error 0.3). However, it also showed nonlinear phases of very rapid individual progression, with an annualized progression reaching up to 5.5 SARA points per year (P2.1) in a cluster of 3 patients, occurring at variable durations of ataxia (for example, in P1, P2.1, and P26 at 5, 10, and 15 years after ataxia onset) (figure 6E).

Using the longitudinal progression estimate, we calculated preliminary sample sizes required to detect different treatment effects in parallel-group interventional trials. To detect a 50% reduction in SARA score progression with 80% power, a total of 330 patients would be required in a 1-year parallel-group trial with visits at 0, 6, and 12 months, and 132 patients would be required in a 2-year parallel-group trial with visits at 0, 12, and 24 months (figure 6F).

## Discussion

This study leveraged 2 independent ataxia cohorts recruited via a large cross-European ataxia network to provide in-depth longitudinal phenotyping and quantitative natural history data on RFC1 disease, including preliminary sample size calculations, thus preparing the first steps toward trial readiness in this novel disease.

Our screening demonstrates that RFC1 disease is frequent across European countries but extends more globally. The 2 novel cohorts independently suggest that RFC1 disease accounts for a substantial share of patients with so far unsolved ataxia, demonstrating this might be the case not only in cohorts expected to be phenotypically enriched for RFC1 disease (cohort A, 67%)^1^ but also for completely unselected “as-comes-in” late-onset ataxia cohorts (cohort B, 14%). This high frequency rate of 14% may apply especially to populations with high consanguinity rates (50% in cohort B), while the prevalence of RFC1 disease in other populations could be closer to 1% to 4%, depending on the region and consanguinity rates.^16,17^ Our results highlight that RFC1 disease is prevalent also in populations without central European origin such as Western Asian populations (Turkish populations, cohort B). Together with the identification of an Indonesian RFC1-family, with even severe disease course and premature death, these findings support the notion of RFC1 as a more global disease extending to Asia.

We show that in patients with unsolved ataxias, ACC and CANVAS are phenotypic clusters of high diagnostic value, helping to flag underlying RFC1 disease, yet as variable clusters along a continuous multisystemic spectrum of RFC1 disease. In fact, *RFC1* was the major gene underlying ACC, accounting for all 29 patients, thus providing the genetic basis of this striking phenotype, which has been reported in the literature both as part of the CANVAS spectrum^4,8,14^ and a distinct syndrome in autosomal-recessive^6^ and autosomal-dominant^18^ ataxia syndromes. The mechanism underlying chronic cough remains to be determined. Dysphagia with recurrent mild aspirations, common in other degenerative ataxias,^19,20^ is unlikely in RFC1 disease; we show that cough occurs on average 26.5 years before the onset of dysphagia. Gastroesophageal reflux, potentially irritating airways, was reported in 31% of RFC1-positive patients but was not associated with cough. With its multisystemic impact, RFC1 disease could damage both peripheral tracts of the cough reflex, for example, by sensory neuropathy of the vagal nerve, and central networks in the brainstem and cerebellum.^21^ As demonstrated here, brainstem and cerebellum are indeed key sites of brain atrophy on MRI in RFC1 disease, and functional network disturbance in these regions might precede their structural decay on routine MRI by decades.

Our findings also confirm *RFC1* as the major gene causing CANVAS, yet the relative share of 68% RFC1-positive patients with clinical CANVAS and of 90% RFC1-positive patients with CANVAS with additional electrophysiologic evidence, respectively, demonstrates that other genes^2^ and nongenetic causes of CANVAS remain to be identified. This notion is supported by the observation of nongenetic toxic CANVAS co-occurring within even the same family as genetic RFC1-associated CANVAS. Indirect evidence for additional genetic causes of CANVAS is provided by the observation of 2 RFC1-negative multiplex CANVAS families (P4.1 and P36).

While ACC and CANVAS have high diagnostic value for underlying RFC1 disease, our findings highlight that they should not be seen as monolithic syndromic entities but rather as 2 instances of variable clusters along a continuous overlapping spectrum of RFC1 disease with variable combinations of 5 recurrent core features: cerebellar ataxia, sensory neuropathy, vestibulopathy, cough, and autonomic dysfunction.

Our in-depth phenotyping highlights that the spectrum of RFC1 disease should indeed be conceptualized far beyond the classic ACC and CANVAS clusters, thereby extending previous phenotypic characterizations of RFC1 disease.^5^ Specifically, we demonstrate that RFC1 disease is predominantly multisystemic beyond CANVAS, including in particular several recurrent features and feature combinations that overlap with and mimic other neurodegenerative diseases. The co-occurrence of ataxia with autonomic dysfunction, bradykinesia, and even features of REM sleep behavior disorder not only overlaps with MSA-C phenotypes but even formally meets the diagnosis criteria of possible MSA^22^ (while RFC1, of course, does not cause pathologically confirmed MSA^23^). This partial overlap of RFC1 disease with MSA-C in at least a subset of patients is additionally supported by the early occurrence of dysphagia (≤10 years after ataxia onset), brainstem atrophy on MRI, early dependence on walking aids, and in particular the rapid MSA-C–like disease progression phases in some participants with RFC1 (5.5 and 2.5 SARA points per year in P1 and P30 with bradykinesia and autonomic dysfunction).

Similarly, the co-occurrence of ataxia with postural instability (49%), early falls (25% before 3 years of ataxia), cognitive impairment (25%), or slowing of vertical saccades (17%) in RFC1 disease shows overlap with probable PSP, especially of cerebellar type.^24^ The multisystemic spectrum of RFC1 disease included also hyperkinetic movement disorders in 11% of patients, comprising mild but continuous dyskinetic or dystonic features and prompting prior sequencing of the Huntington disease gene in 2 of the dyskinetic patients with RFC1 who additionally showed slowed horizontal saccades.

Our observation of frequent cognitive impairment in patients with RFC1 (25%) indicates the need for future in-depth neuropsychological studies, mapping systematically the neuropsychological profile of RFC1 disease, which will need to include, in particular, cognitive-affective features as often observed in cerebellar disease.^25^

Unidentified RFC1 disease is still frequent among patients with unsolved ataxia, as indicated by our 2 screening cohorts, highlighting the need for additional clinical and genetic identification strategies. We performed here the first systematic analysis of clinical features that might allow discriminating RFC1-positive from RFC1-negative ataxia. Afferent ataxia (i.e., marked worsening of gait ataxia without visual control), sensory symptoms, and chronic cough not only were more common in RFC1-positive patients but also yielded a positive predictive value of >90%. These features might thus help to clinically indicate underlying RFC1 disease, in particular if occurring in combination.

In addition, unidentified patients with RFC1 might also be successfully detected by WGS and, as indicated by our proof-of-principle application of a novel search algorithm, even by WES analysis, despite the intronic location of *RFC1* repeat expansions. While *RFC1* repeat expansions have previously been reidentified by WES on the basis of its prior identification by WGS,^2^ our approach was indeed performed as an unbiased approach without prior knowledge of the existence of the RFC1 repeats, that is, without prior WGS, and independently from and blinded to the PCR-based screening. The prioritization of RFC1-positive cases in 2 exemplary WES datasets is particularly remarkable because it exploits off-target reads. There was a low a priori chance to gain crucial information for a given off-target position in WES, with only ≈25% of reads mapping into intronic or intergenic regions that comprise at least 98.5% of the human genome. If confirmed by future systematic validation studies, this WES/WGS–based identification strategy of *RFC1* repeat expansions might be applicable as a first-line screening strategy for existing WES/WGS datasets of unsolved ataxia cohorts, thus saving resources and allowing accelerated identification of still unidentified patients with RFC1.

Our study provides semiquantitative and quantitative data on progression of RFC1 disease and leverages longitudinal data to further elaborate cross-sectional findings. Semiquantitative capture of functional disease progression by SDFS score demonstrates substantial interindividual variability in functional impairment, confirming and specifying nonquantitative observations from a prior RFC1 cohort.^5^

Capture of ataxia progression in RFC1 disease by the SARA score reveals an average progression of 1.3 SARA points per year according to cross-sectional estimates, which is confirmed by our longitudinal data. Thus, ataxia progression in RFC1 disease is almost twice as fast as in Friedreich ataxia (0.77 SARA points per year^13^), which represents the most common autosomal-recessive ataxia together with RFC1 ataxia and is likewise a mixed afferent-cerebellar ataxia. Our longitudinal data additionally reveal that individual RFC1 disease progression can include sudden phases of rapid, MSA-C–like ataxia progression up to 5.5 SARA points per year and that such phases of accelerated progression can occur after variable disease duration (5–15 years). This intraindividual variability in disease progression is mirrored by an also substantial interindividual variability in disease progression, with some patients showing the same degree of ataxia severity even with a difference of >10 years’ disease duration.

In addition, the evolution of CANVAS features observed in this cohort indicates that RFC1 disease does not follow a uniform systems spread of disease but rather interindividually relatively variable hits of cerebellar, sensory, or vestibular damage with variable progression dynamics within each system due to individual genetic and nongenetic (e.g., toxic) modifiers. Larger longitudinal long-term RFC1 studies are warranted to confirm this hypothesis.

Limitations of this study are its relatively small cohort size in the longitudinal study part and the lack of volumetric MRI and other quantitative fluid and digital-motor biomarkers. However, it presents a starting point for hypotheses that will guide more in-depth longitudinal analysis and future trials in this highly prevalent, newly identified disease. The observation of a broad multisystemic disease spectrum of RFC1 disease suggests that both future natural history trials and in particular treatment trials need to include multimodal or composite outcome measures, allowing capture of the multisystemic, interindividually variable phenotypic spectrum and the individual's differential systems dynamics and response over time. The relatively high numbers in our preliminary sample size estimations, with 132 patients in a 2-year trial, reflect the need for future trials to be sufficiently powered to accommodate the partly nonlinear, accelerated intraindividual progression of RFC1 disease demonstrated here.

## Glossary

ACC: ataxia with chronic cough
CANVAS: cerebellar ataxia, neuropathy, and vestibular areflexia syndrome
CI: confidence interval
IQR: interquartile range
MSA-C: cerebellar-type multiple system atrophy
NPV: negative predictive value
PPV: positive predictive value
PSP: progressive supranuclear palsy
RFC1: replication factor complex subunit 1
SARA: Scale for the Assessment and Rating of Ataxia
SDFS: spinocerebellar degeneration functional scale
WES: whole-exome sequencing
WGS: whole-genome sequencing
